# Test and Improvement of a Fuze MEMS Setback Arming Device Based on the EDM Process

**DOI:** 10.3390/mi13020292

**Published:** 2022-02-12

**Authors:** Yu Qin, Yanbai Shen, Xiannan Zou, Yongping Hao

**Affiliations:** 1 School of Resources and Civil Engineering, Northeastern University, Shenyang 110819, China; shenyanbai1@mail.neu.edu.cn; 2 School of Mechanical Engineering and Automation, Northeastern University, Shenyang 110819, China; zxn673436468@163.com; 3 School of Mechanical Engineering, Shenyang Ligong University, Shenyang 110159, China; yongping21@126.com

**Keywords:** MEMS S&A device, fuze, microspring, EDM process, setback slider

## Abstract

This paper introduces the working principle of a MEMS safety and arming (S&A) device for a fuze that is installed perpendicular to the axis of the projectile. Additionally, the application of low-speed wire electrical discharge machining (EDM) in the fabrication of the device is proposed. Microsprings are susceptible to flexural deformation and secondary deformation in the EDM process, a problem that is solved by designing the auxiliary support beam, using multiple cuts, destress annealing and optimizing the processing parameters. The difficult problem of setback slider deformation in the principle prototype test is properly solved by establishing V-shaped grooves at both ends of the setback slider. The connection mode between the microspring and the frame is changed to a clearance fit connection. The improved setback arming device can guarantee service process safety and launch reliability. The maximum overload that can be withstood in service processing is 20,000 g, and the minimum overload for safety release during launch is 12,000 g. The results show that the EDM process can greatly reduce the machining cost while improving the machining precision and machining speed, which can compensate for the defects of the current manufacturing technology.

## 1. Introduction

**Figure 1:**
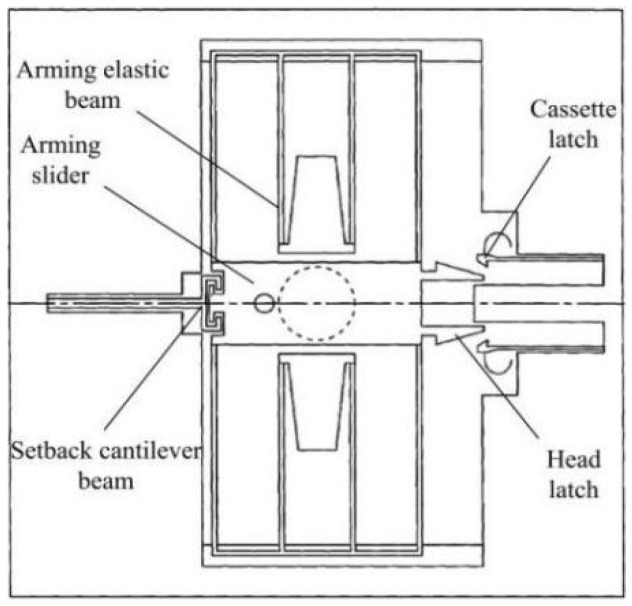
Structure designed by Koehler.

In recent years, with the development of intelligent ammunition technology, modern fuzes have been endowed with more functions, such as flight control, explosion point control, trajectory correction, target recognition, and positioning, in addition to state control and initiation control. However, the volume limitations of traditional fuzes restrict the expansion of these functions, and this fuze-development problem has been effectively solved by the emergence of MEMS fuzes [1,2,3,4,5]. A setback arming device is the core device in a MEMS fuze and is used to ensure service process safety and launch reliability [6,7,8,9,10]. Most setback arming devices are oriented parallel to the projectile axis when installed, meaning that the explosive train is not in the same straight line [11,12,13]. Therefore, the direction of detonation energy transmission needs to be changed, resulting in energy loss. In serious cases, the detonator may not be able to detonate the warhead charge, resulting in a “dud” phenomenon [14,15]. To create MEMS fuzes with higher performance and more space to achieve additional functions, research on MEMS setback arming devices that are placed perpendicular to the projectile axis (hereinafter referred to as “vertical-frame-based setback arming devices”) has gradually become popular in recent years [16,17,18,19,20]. Figure 1 shows a vertical-frame-based setback arming device that was proposed by Koehler et al. [21], which consists of a setback cantilever beam, an arming elastic beam, an arming slider, a head latch, and a cassette latch. Generally, the arming slider is locked in the safe position by the setback cantilever beam and the arming elastic beam. When a projectile is launched, the setback cantilever beam moves downward under the action of the setback force and breaks away from the arming slider, thus removing the restriction on the arming slider.

**Figure 2:**
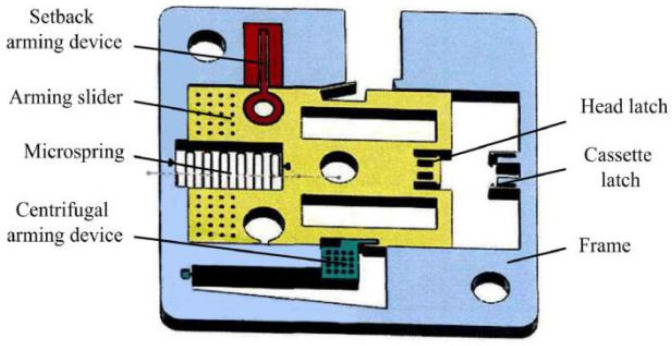
Structure designed by Li.

**Figure 3:**
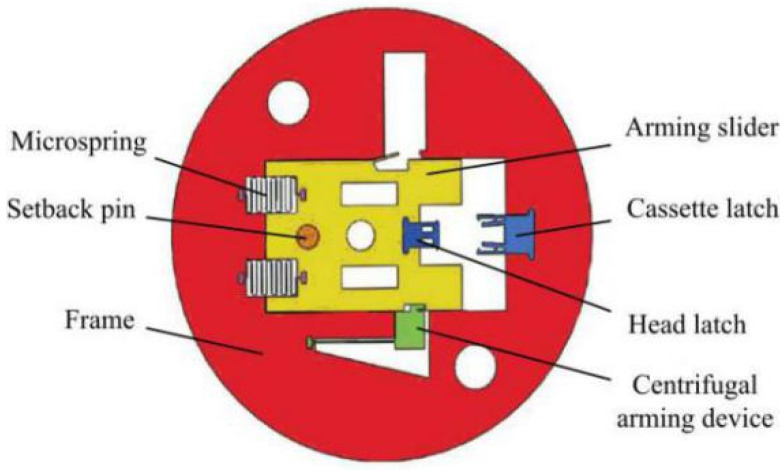
Structure designed by Tong.

Figure 2 is a cantilever lock setback arming device proposed by Li [22], which is installed perpendicular to the axis of the projectile. The response characteristics of the device under the two acceleration environments of service processing and normal launch are analyzed theoretically and verified by simulation. The results show that the cantilever lock setback arming device can effectively distinguish service processing from launch. Tong [23] improved the design on this basis and proposed a microinertial pin setback arming device, as shown in Figure 3. The scheme with a tilted microinertia pin was adopted to effectively reduce the axial dimension of the device, and the feasibility of the device was verified by theoretical calculations and simulation analysis.

The structures designed by Koehler [21], Li [22] and Tong [23] all use environmental forces to release the safety. The research mainly focuses on theoretical calculations and simulation analysis, lacking effective test verification, and does not involve research on the manufacturing process. For the fabrication of MEMS setback arming devices, nonsilicon-based micromachining technology (generally a lithography electroforming micro molding (LIGA) process or an ultraviolet-LIGA (UV-LIGA) process) and silicon-based micromachining technology (generally a deep reactive-ion etching (DRIE) process or a silicon-on-insulator (SOI) process) are commonly used. The cost of these processes is high, and no cheap mass production method has been found [24,25]. To solve the above problems, a vertical-frame-based setback arming device that also uses environmental force to release the safety is proposed in this paper, and low-speed wire electrical discharge machining (EDM) is successfully applied to the fabrication of the device. The principle prototype has the advantages of high precision and low cost. A mechanical impact test and centrifugal overload test were carried out on the principle prototype, and its structure was subsequently improved according to the problems identified in the tests. The improved principle prototype can accurately identify the service processing and launch stages. The results provide technical support for promoting the application and popularization of MEMS fuzes in the field of intelligent ammunition.

## 2. Working Principle of the Setback Arming Device

**Figure 4:**
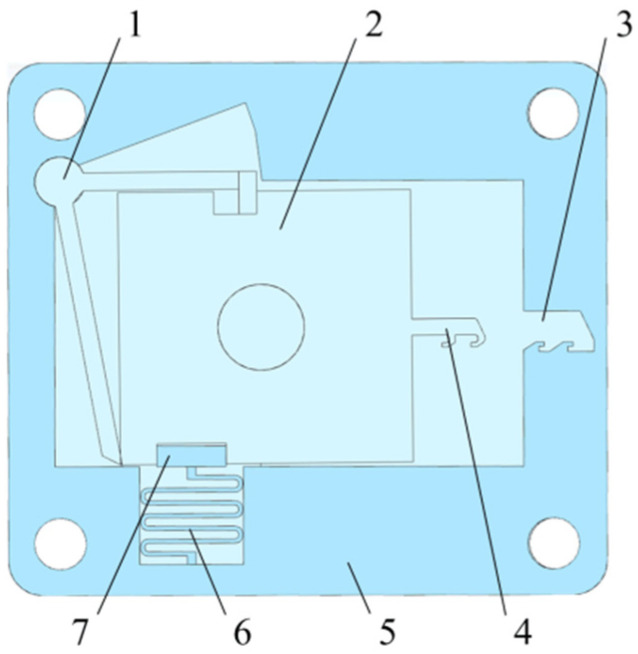
Vertical-frame-based setback arming device. 1—Driving wheel, 2—Arming slider, 3—Cassette latch, 4—Head latch, 5—Frame, 6—Microspring and 7—Setback slider.

**Figure 5:**
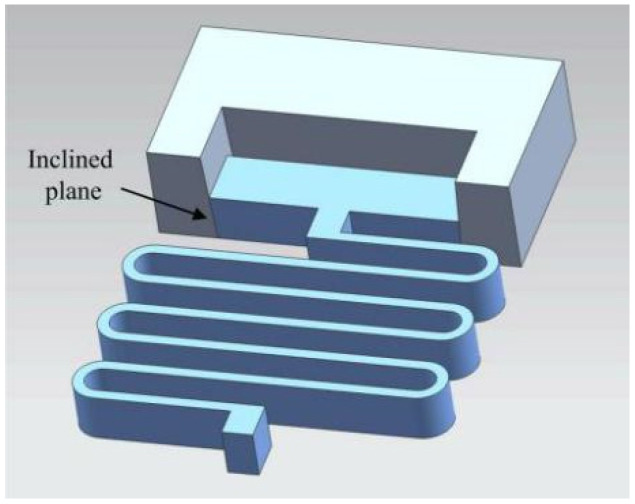
The inclined plane of the setback slider.

**Figure 6:**
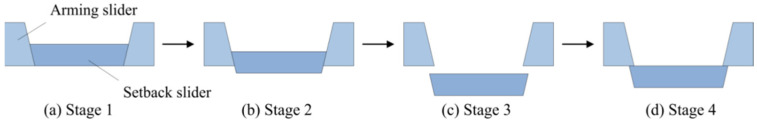
Schematic diagram of the safety release.

Figure 4 shows a MEMS safety and arming (S&A) device applied to a certain type of 105 mm grenade, and its size is 12 mm × 10 mm × 0.8 mm. When the device is installed, it is oriented perpendicular to the axis of the projectile, and it mainly includes a setback arming device and arming device. The setback arming device is composed of a setback slider, a microspring, and a frame, and the arming device is composed of a driving wheel, a pin pusher, an arming slider, a head latch, and a cassette latch. The setback arming device is mainly studied in this paper. The application background of the requirements is as follows: the peak value of the most dangerous drop overload in the service processing stage is 18,000 g, the duration is 0.1 ms, the peak value of the launch overload is 12,000 g, and the duration is 9 ms. This kind of setback arming device has the advantage of a simple structure and can be used to replace the setback slider and the zigzag slot in the traditional device with a pair of inclined setback sliders and arming sliders. In the initial state, the setback slider and the arming slider are tightly assembled together and connected to the frame with microsprings. The ANSYS/LS-DYNA finite element software simulation shows that when the slope angle of the setback slider and the arming slider is set to 87°, the service processing and launch stages can be distinguished by controlling the movement time of the setback slider, as shown in Figure 5. The service processing action time is very short, and the inertial force induced on the setback slider is not high enough to overcome the friction between the inclined surfaces. When the inertial force disappears, the setback slider returns to its original position under the action of the microspring restoring force. The launch action time is relatively long. The setback slider moves away from the arming slider under the action of a continuous setback force. In the process of movement, the setback slider is squeezed by the arming slider, undergoes a deflection deformation, and returns to its original shape after passing through the inclined plane. At this time, the setback slider cannot return to the initial position due to the obstruction of the arming slider, and the process of safety release is shown in Figure 6.

## 3. Processing the Setback Arming Device

### 3.1. Process Selection

**Figure 7:**
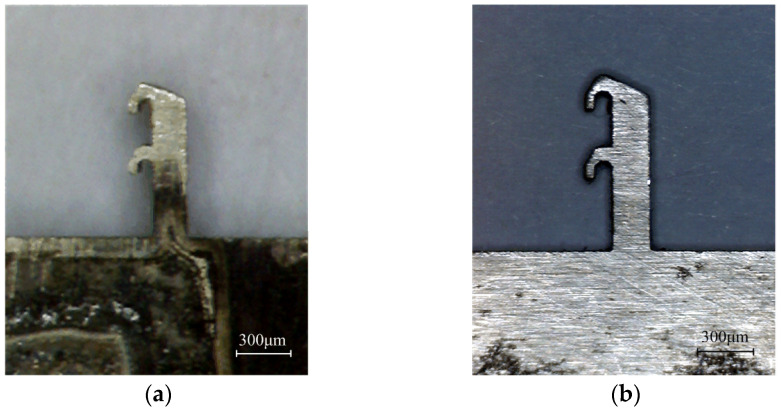
A head latch. (**a**) UV-LIGA process; (**b**) EDM process.

The difficulties in the fabrication of the setback arming device are mainly related to two aspects. On one hand, the thickness of the microspring is 0.4 mm, while the thickness of the frame is 0.8 mm, and the microspring and the frame with different thicknesses need to be processed into a composite device. On the other hand, the contact surface between the setback slider and the arming slider is not a plane but needs to be processed into an inclined plane. The UV-LIGA process has the highest technical maturity among current nonsilicon-based micromachining technologies, while the EDM process has unique advantages in the fabrication of microparts [26,27,28,29,30]. Figure 7 shows a head latch of the MEMS S&A device fabricated by the UV-LIGA process and the EDM process, using 10 of each for process comparison, and the results are shown in Table 1. It can be seen from the table that the EDM process has the advantages of high precision, high speed, and low cost. Therefore, the EDM process is applied to manufacture the setback arming device through the technical research in this paper, and the combined electrode fixture is used to enable simultaneous processing of multiple electrodes. This method can be used to greatly reduce the machining cost while improving the machining precision and machining speed.

### 3.2. Processing Scheme

**Figure 8:**
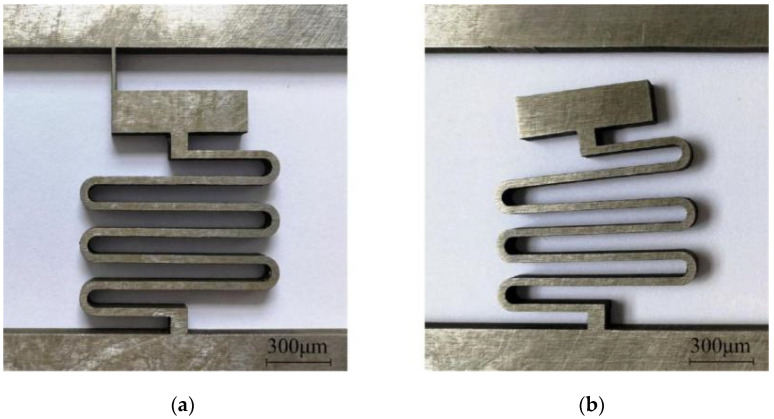
Processing scheme for the support beam width of 0.06 mm. (**a**) Before (**b**) After.

Microspring machining is technically difficult in the fabrication of setback arming devices by EDM; this is the main reason why the EDM process cannot be used in setback arming device manufacturing. Due to the size effect and residual stress, microsprings are prone to flexural deformation and secondary deformation in the machining process. In this paper, a high-purity hard nickel plate is selected as the processing material, and the processing machine is a CA30 immersion precision CNC low-speed wire cutting machine produced by Agie Charmilles Company (Biel, Switzerland). The processing scheme that involves orienting the auxiliary support beam in the longitudinal direction of the microspring is adopted, as shown in Figure 8a. After a trial cutting of the microspring, it is found that when the width of the support beam is set to 0.06 mm, the processed microspring undergoes a large deflection deformation and deviates to one side of the support beam, as shown in Figure 8b. On one hand, this phenomenon may be due to the long EDM discharge time that is required when the support beam with this width is removed, leading to a longer time during which the flushing pressure and discharge reaction force act on the microspring and excessive residual stress after machining. On the other hand, this phenomenon may be related to the position of the support beam. The support beam is located on one side of the microspring end, providing an uneven support force that results in a stress concentration when the support beam is removed.

**Figure 9:**
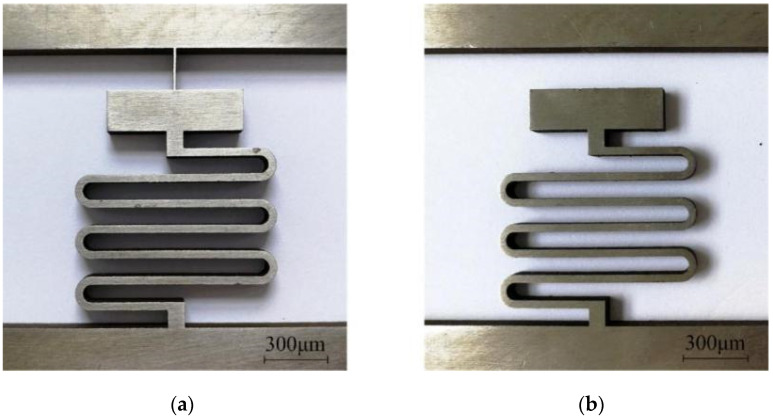
Processing scheme for the support beam width of 0.04 mm. (**a**) Before; (**b**) After.

To reduce the flexural deformation in the machining process, a method of increasing the pulse interval, reducing the peak current, and reducing the flushing pressure was proposed in reference [31] to reduce the reaction force during EDM discharge. After trial cutting, it is found that the deflection deformation of the microspring can be improved to a certain extent by adjusting the processing parameters, but the effect is not evident. Therefore, this paper adopts the method of optimizing the processing parameters through energetic control and changing the processing scheme to reduce the deflection deformation. The processing parameters used are shown in Table 2. Figure 9a shows the modified processing scheme, in which the support beam is set at the center of the microspring end, and the width is reduced to 0.04 mm. With this scheme, the action time of the EDM process decreases when the support beam is removed, the deflection deformation of the microspring after processing is very small and negligible, and the shape accuracy of the microspring is good without inclination, as shown in Figure 9b.

**Figure 10:**
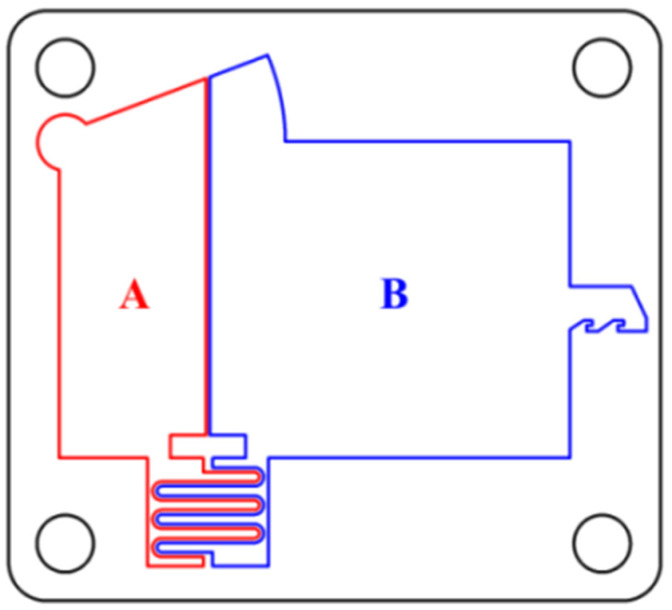
Frame processing scheme.

**Figure 11:**
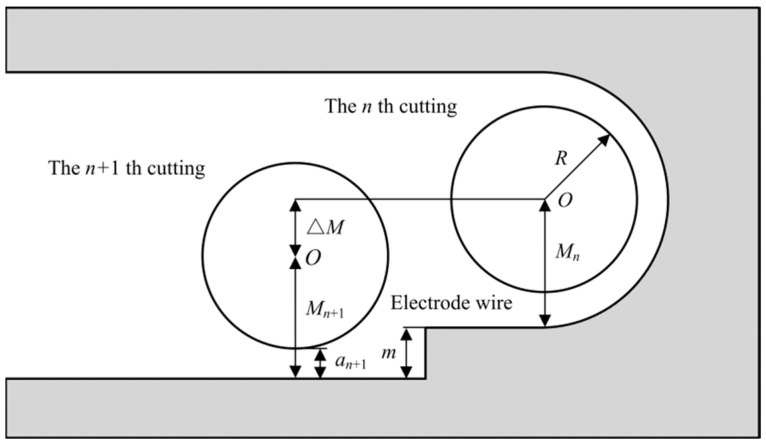
A schematic diagram of multiple cutting.

Figure 10 shows the processing scheme of the frame. The auxiliary support beam with a width of 0.04 mm divides the frame into two processing areas, A and B. Before processing, bolt holes are machined around the frame with an EDM prepiercing machine, and different machining allowances and parameters are selected to produce multiple cuts between A and B according to the cutting path planned in the scheme. This method can not only be used to improve the quality of the processing surface but also to redistribute the residual stress and correct the deflection deformation of the microspring. In this paper, a total of five cuttings were performed. After the first cutting, the surface quality of the frame was poor, and many adhesives were attached. This is because the first cutting is rough machining, the discharge energy is large, and the frame surface is seriously ablated. In addition, due to the size effect, the flushing pressure during cutting cannot be too large, the adhesive cannot be effectively removed, and the surface roughness Ra is 1.43 μm. Figure 11 is a schematic diagram of multiple cutting, the relationship of the size is:

(1) $$M_{n}=M_{n+1}+\Delta M-m$$

(2) $$M_{n+1}=a_{n+1}+R$$

*M_n_* is the offset of the electrode wire at the *n* th cutting; *M_n__+_*_1_ is the offset of the electrode wire at the *n* *+* 1 th cutting; Δ*M* is the offset increment after two adjacent cuts; *m* is the surface height difference after two adjacent cuts, which can be measured directly; *R* is the radius of electrode wire, brass wire with a diameter of 100 μm was selected; *a_n__+_*_1_ is the unilateral discharge gap at the *n* th cutting, and the unilateral discharge gap for the five cuttings are shown in Table 3. Since the offset of the electrode wire does not correspond to the given value of the machine tool, it is necessary to correct the offset of the electrode wire one by one during multiple cutting. To ensure the machining accuracy, the offset of the electrode wire in the last cutting should be the sum of the unilateral discharge gap and the electrode wire radius. For the limitation of discharge gap, machining allowance, and machining space, the corrected electrode wire offset is shown in Table 4. The final surface roughness Ra can reach 0.49 μm after five cuttings with the offset, which is reduced by 65.7% compared with the first cutting.

Due to the small size of the inclined planes on both sides of the setback slider, if two inclined planes are processed at the same time, the requirements for preparing the formed electrode are higher. Therefore, the method for separately processing each single inclined plane is adopted in this paper. The surface to be processed is fixed at an incline during processing, and the bottom and side of the electrode wire were used for processing. The frame with an auxiliary support beam was then heated to 600 °C in a two-chamber vacuum permeable furnace for destress annealing treatment. After two hours, the device was placed in air to naturally cool [32]. After this step, the residual stress of the microspring was smaller, and there was no secondary deformation due to the high temperature during processing. Finally, the auxiliary support beam was removed using the EDM process.

**Figure 12:**
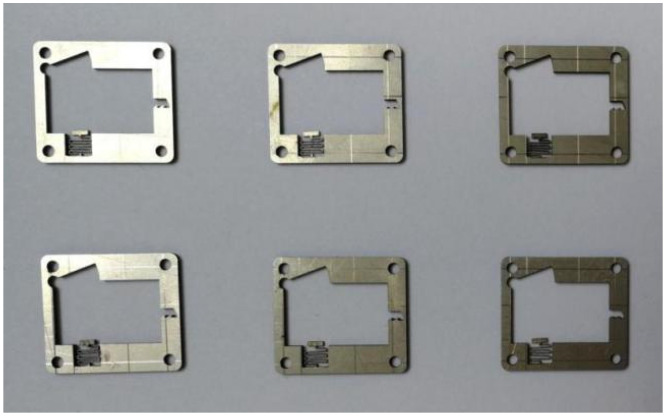
Microspring and frame samples.

**Figure 13:**
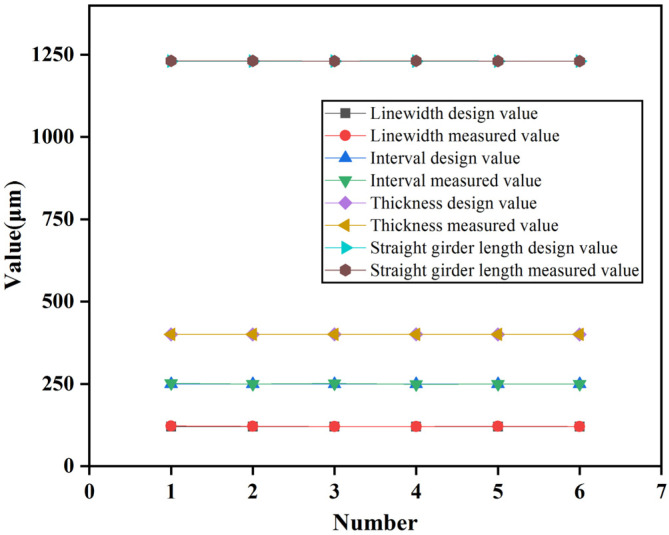
The measurement results.

**Figure 14:**
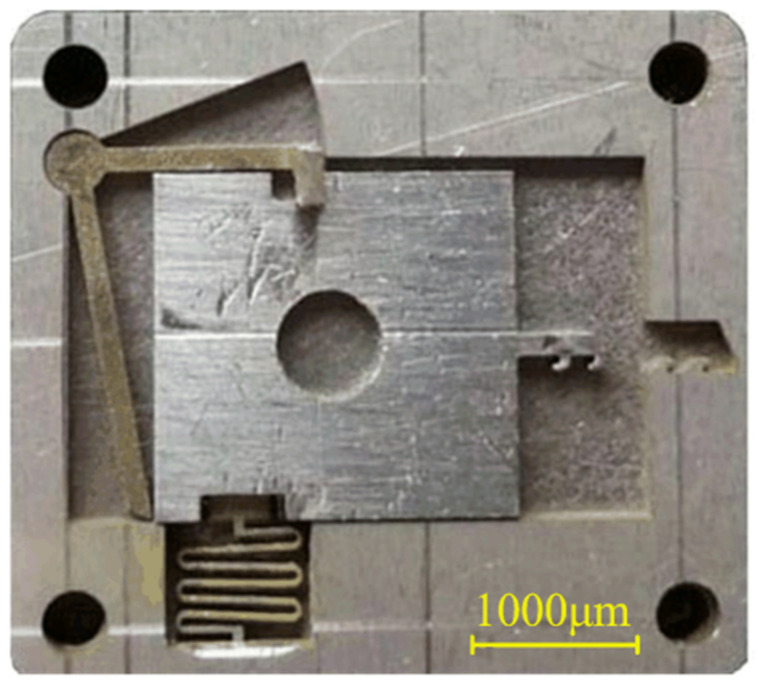
Principle prototype.

Figure 12 shows the sample comprising the microspring and frame after processing. This figure shows that the microspring has a small flexural deformation and good consistency. The microspring and setback slider were measured after processing with stereomicroscopy. The measurement results are shown in Figure 13. The measured values of the main parameters are similar to the design values, indicating that the EDM processing scheme proposed in this paper has high precision and can meet the processing requirements. The principle prototype obtained after assembly is shown in Figure 14.

## 4. Testing the Setback Arming Device

According to the regulations in GJB573A-1998 “Fuse Environment and Performance Test Method”, the setback arming device needs to endure mechanical impact tests and centrifugal overload tests.

### 4.1. Mechanical Impact Test Results

A servo vertical impact testing machine was used for the mechanical impact test, and 10 principle prototypes were used for testing. A half sinusoidal pulse with a duration of 200 μs and a peak value of 100 μs was used to simulate the setback acceleration environment during service processing. The test results show that when the peak acceleration decreased from 23,000 g to 14,000 g, the setback slider remained in the initial position, and the safety of the setback arming device was not released. The setback arming device can guarantee the safety of service processing.

### 4.2. Centrifugal Overload Test Results

A rotating arm centrifugal testing machine was used for the centrifugal overload test, and 10 principle prototypes were used for testing. The centrifugal force generated by rotation was used to simulate the setback force during launch. The test results show that when the centrifugal acceleration decreased from 17,000 g to 8000 g, the setback slider remained in the initial position, and the safety of the setback arming device was not released. The setback arming device cannot guarantee the reliability of the launch. This phenomenon may be due to the large bending deformation of the setback slider when the setback arming device is released, where the acceleration required for deformation is very large, so the test conditions cannot meet the deformation requirements.

## 5. Improvement of the Setback Arming Device

The results of the mechanical impact test and centrifugal overload test show that the existing setback arming device cannot effectively distinguish service processing from launch, and its structure needs to be improved.

### 5.1. Improvement Scheme of Setback Slider

**Figure 15:**
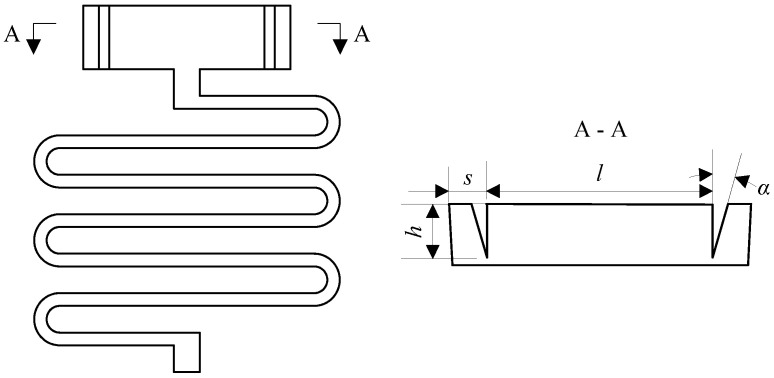
Improvement scheme for the setback slider.

To solve the difficult problem of the setback slider deformation in the setback arming device, a scheme including V-shaped grooves at both ends of the slider can be used for improvement. On the premise of ensuring the safety of service processing, the acceleration required for flexural deformation of the setback slider during launch can be reduced. As shown in Figure 15, the reasonable selection of the distance *s* from the vertical sidewall to the inclined plane, the distance *l* between the vertical sidewalls, the vertical sidewall depth *h*, and the sharp angle *α* of the two V-shaped grooves are the key factors for enabling the setback arming device to meet the design requirements.

In this paper, ANSYS/LS-DYNA finite element software is used to simulate the movement of the setback slider in the launch and service processing stages by adjusting the distance *s* from the vertical sidewalls of the two V-shaped grooves to the inclined plane, the distance *l* between the vertical sidewalls, the depth *h* of the vertical sidewalls and the sharp angle *α*. Through these simulations, the reasonable ranges for *s*, *l*, *h*, and *α* can be found. The specific steps are as follows: the distances *s* and *l* are gradually increased and the depth *h* and angle *α* are gradually decreased in the launch stage until the setback slider is able to completely release the safety. At this time, *s*, *l*, *h*, and *α* are at their minimum values. The distances *s* and *l* are gradually decreased and the depth *h* and angle *α* are gradually increased in the service processing stage until the setback slider returns to the initial position. At this time, *s*, *l*, *h*, and *α* are at their maximum values. The simulation results show that to ensure that the setback slider can be released completely in the launch and return to the initial position in service processing, the range of the distance s from the vertical sidewall of the V-shaped groove to the inclined plane should be 0.2~0.3 mm, the range of the distance l between the vertical sidewalls should be 1~2 mm, the range of the depth *h* of the vertical sidewall should be 0.3~0.4 mm, and the range of the sharp angle *α* should be 15.5°~16.5°. Therefore, the values *s* = 0.25 mm, *l* = 1.5 mm, *h* = 0.35 mm, and *α* = 16° are preliminarily selected.

### 5.2. Simulation of the Improved Setback Slider

#### 5.2.1. Simulation of Service Processing

**Figure 16:**
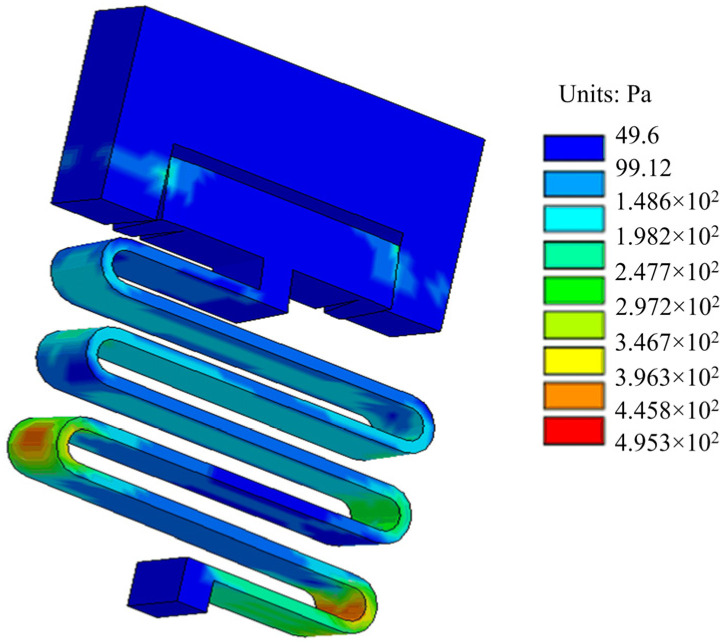
Stress distribution in service processing.

**Figure 17:**
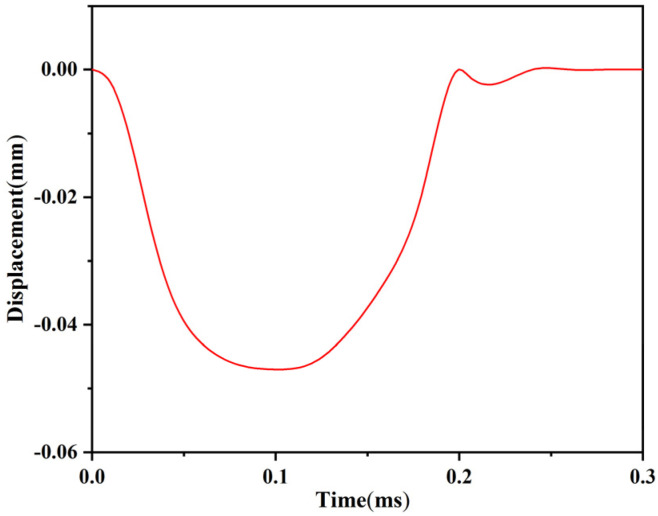
Time–displacement curve of the setback slider in service processing.

The setback load in service processing is set to 18,000 g in the ANSYS/LS-DYNA software. To observe the displacement of the setback slider after the load is removed, the simulation time is set to 0.3 ms. Figure 16 shows the stress distribution nephogram of the setback arming device at 0.1 ms. It can be seen from the figure that the angle of the V-shaped groove at both ends of the setback slider decreases, but it does not break away from the arming slider. The maximum stress of the setback arming device in the figure is 495.3 Pa, which is less than the allowable stress for a nickel material, so the material does not undergo plastic deformation. Figure 17 shows the time–displacement curve of the setback slider in service processing. The displacement of the setback slider increases rapidly after the movement begins, reaching a maximum value of 0.047 mm at 0.1 ms. When the load is removed, the displacement decreases with increasing time, returning to 0 from 0.24 ms and then remaining unchanged; this indicates that the setback slider returns to the initial position under the action of the microspring restoring force.

#### 5.2.2. Simulation of the Launch

**Figure 18:**
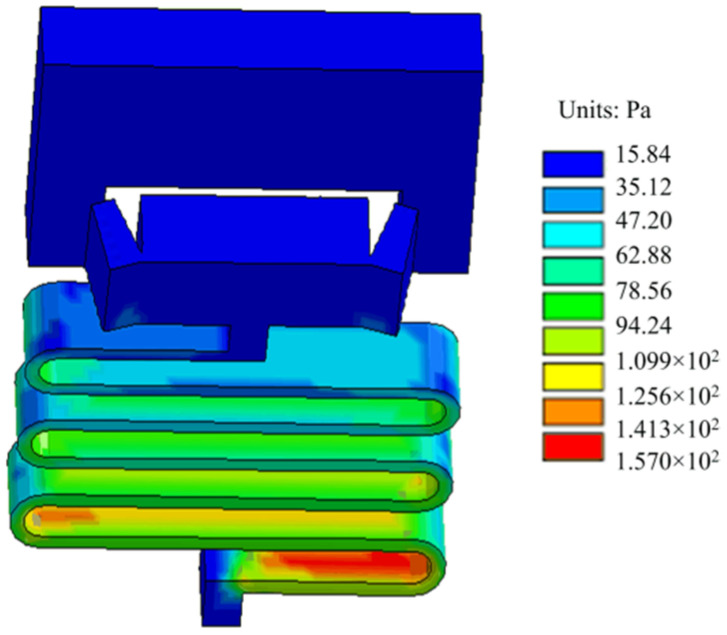
Stress distribution in the launch stage.

**Figure 19:**
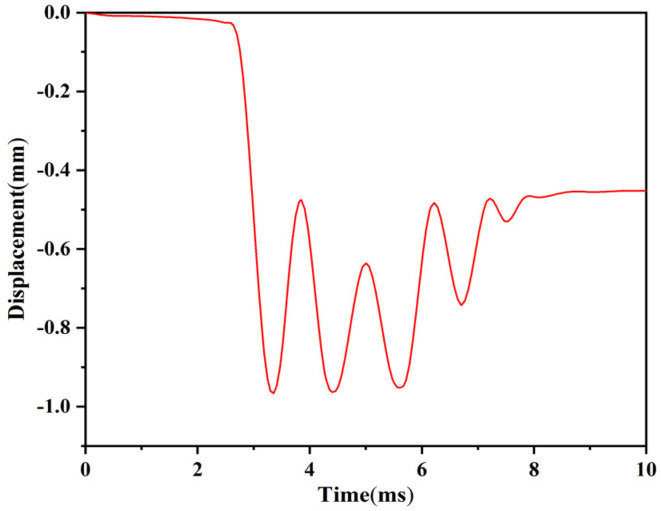
Time–displacement curve of the setback slider at launch.

The setback load at launch is set to 12,000 g, and the simulation time is set to 10 ms. The setback slider moves away from the arming slider under the action of the load, approaches the arming slider under the action of the microspring restoring force, and collides with the obstructing inclined plane of the arming slider. After the collision, the setback slider moves away from the arming slider again under the action of the inertial force, and this process is repeated until the inertial force energy is completely exhausted. Finally, the angle of the V-shaped groove at both ends of the setback slider becomes larger, and it gets stuck on the arming slider. Figure 18 shows the stress distribution nephogram of the setback arming device at 10 ms. The maximum stress value in the figure is 157 Pa, which is less than the allowable stress of a nickel material. Figure 19 shows the time–displacement curve of the setback slider at launch. The figure shows that the displacement of the setback slider increases slowly at the beginning of the movement process, the setback slider starts to move away from the arming slider after 2.75 ms, and the displacement increases rapidly with time, reaching the maximum value of 0.963 mm at 4.4 ms. Additionally, the displacement of the setback slider produces an oscillation curve under the actions of the inertial force and microspring restoring force. After 9.9 ms, the displacement is stable at 0.452 mm and remains unchanged, indicating that the setback arming device is completely released.

The above simulation results show that the improved design of the setback arming device solves the difficult problem of setback slider deformation, achieves the goal of delay arming, and can reliably distinguish service processing from launch.

#### 5.2.3. Processing the Improved Setback Slider

**Figure 20:**
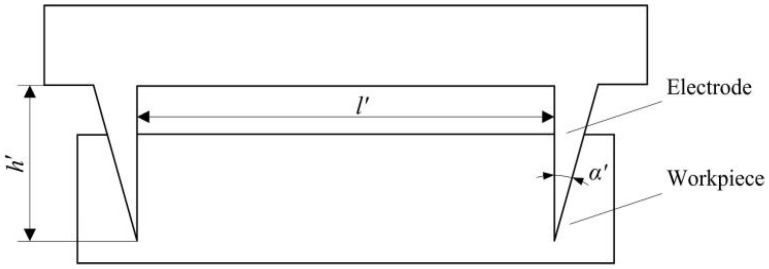
Processing scheme for the V-shaped groove.

According to the improvement scheme of the setback slider, two V-shaped grooves with a vertical sidewall depth of 0.35 mm, a vertical sidewall distance of 0.15 mm, and a sharp angle of 16° need to be processed on a setback slider with a thickness of 0.4 mm. Due to the special shape of the V-shaped groove and the strict requirements of dimensional accuracy and position accuracy, it is difficult to use wire EDM (WEDM), and EDM is generally used instead. EDM has unique advantages for processing precision structures such as narrow slits and sharp grooves [33,34]. This method uses forming electrodes to process workpieces, so it is necessary to prepare special electrodes for processing V-shaped grooves. To ensure the dimensional accuracy and positional accuracy of the V-shaped groove and improve the processing efficiency, the electrode shapes are designed to be two triangles that correspond to the V-shaped groove. The distance between these two electrodes should be equal to the distance between the vertical sidewalls of the V-shaped groove. In addition, to prevent the discharge phenomenon on the connection surface between the electrode and the workpiece, the height of the electrode should be appropriately increased. The processing scheme of the V-shaped groove is shown in Figure 20. In the figure, the distance between the two electrodes is *l*′ = *l* = 1.5 mm, the electrode height is *h*′ = 0.9 mm, and the sharp angle is *α*′ = *α* = 16°.

#### 5.2.4. Electrode Processing

**Figure 21:**
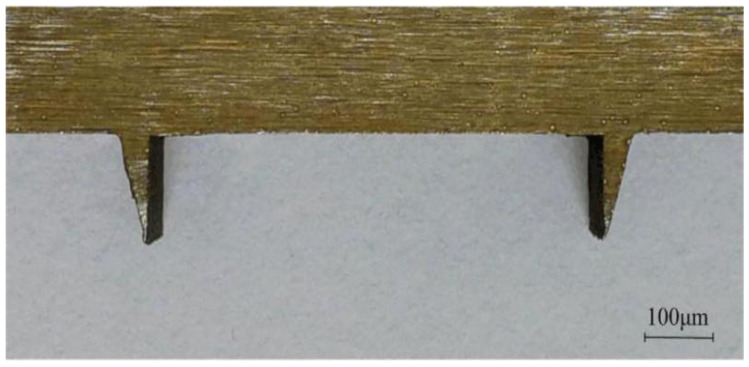
Actual electrode.

The processing accuracy of the electrode directly affects the processing accuracy of the V-shaped groove. To reduce the influence of electrode loss on the processing accuracy, the V-shaped groove forming electrode was fabricated through the WEDM process. A CA30 immersion precision CNC low-speed wire cutting machine tool was used for processing. The electrode material was a copper-tungsten alloy. The copper-tungsten alloy electrode has the characteristics of high-temperature resistance, arc ablation resistance, high electrical conductivity, and high thermal conductivity. The electrode loss during processing was extremely low, which was conducive to improving the quality of the processed surface. The electrode wire was a brass wire with a diameter of 0.2 mm, the dielectric was deionized water, and the resistivity was 1 × 10^5^ Ω·cm. During electrode processing, the machining tool uses a lower feed speed and keeps the speed constant for multiple cuttings. The main parameters are shown in Table 5. Figure 21 shows an actual picture of the processed electrode; the picture shows that the electrode has a good processing quality and high shape accuracy. The processed electrodes were measured via stereoscopic microscopy. The measured results are shown in Table 6. The distance *l*′, height *h*′, and angle *α* between the left and right electrodes are similar to the design values, meeting the processing requirements.

#### 5.2.5. V-Shaped Groove Formation

**Figure 22:**
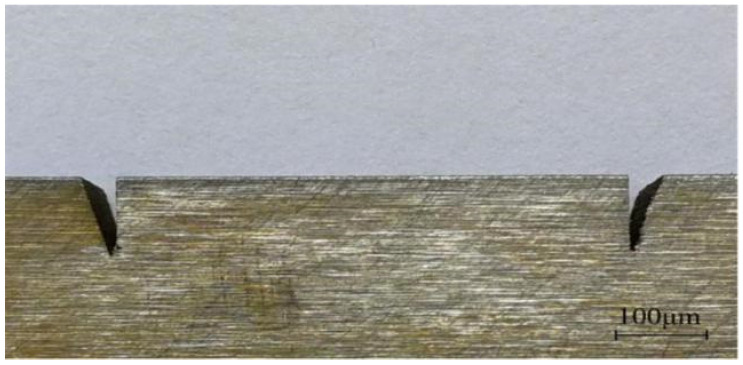
Actual V-shaped groove.

The V-shaped groove was fabricated through the EDM process, and the machining tool used was an EA12AM CNC ultraprecision mirror spark machine produced by Mitsubishi Electric Automation Co., Ltd. (Tokyo, Japan) Figure 22 shows the actual picture of the V-shaped groove after processing; the picture shows that the verticality and shape accuracy of the V-shaped groove sidewall are increased, and there are fewer attachments on the processed surface. The formed V-shaped grooves were measured via stereoscopic microscopy, and the measured results are shown in Table 7. The distance *l*, depth *h*, and angle *α* between the vertical sidewalls of the left and right V-shaped grooves are close to the design values, meeting the processing requirements.

### 5.3. Improvement Scheme for the Microsprings

**Figure 23:**
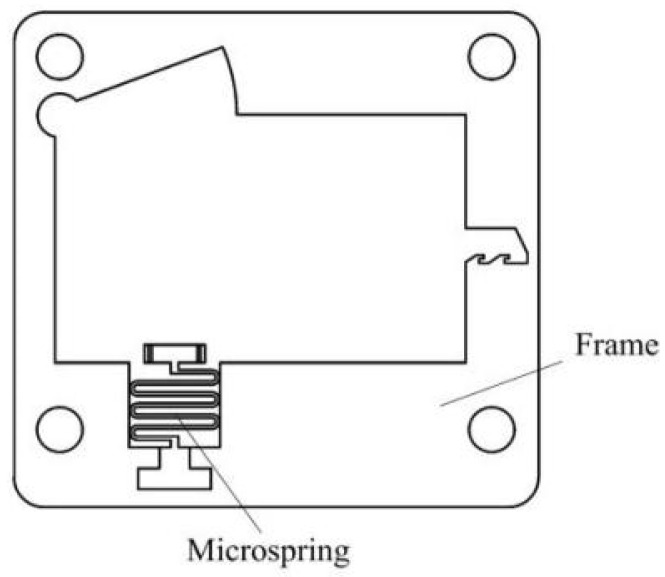
Improved scheme.

In the existing structure, the microspring and the frame are connected as a whole, which produces some inconvenience in the processing stage. Additionally, the frame cannot be reused in the test process, increasing the test cost. Considering that the frame is installed perpendicular to the axis of the projectile, there is ample space in the direction of the frame plane. Therefore, the microspring and the frame can be separated in the design, and a T-shaped mass block is left at the bottom of the microspring. Moreover, the bottom width of the frame is increased, a T-shaped groove corresponding to the size of the mass block is maintained, and the two components are connected through clearance fitting. Figure 23 shows an improved design scheme in which the frame can be processed directly, greatly simplifying the fabrication process.

### 5.4. Processing the Improved Setback Arming Device

**Figure 24:**
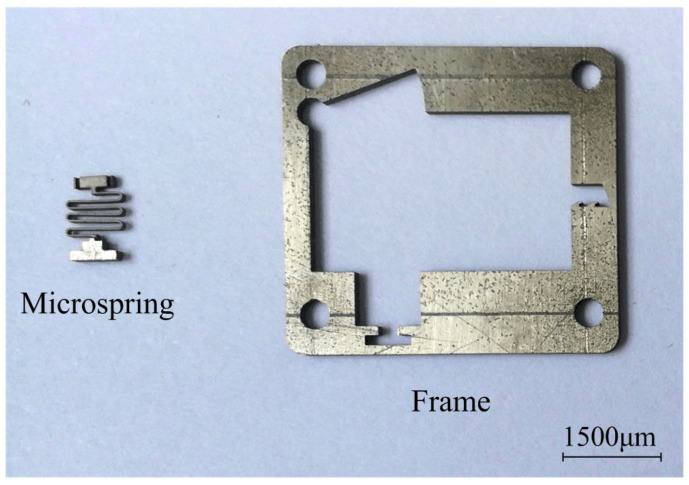
Improved microspring and frame sample.

The processing accuracy of the microspring and V-shaped groove is the key factor for ensuring the safety and launch reliability of the setback arming device. In addition, there is a clear positional relationship between the microspring, the V-shaped groove, and the setback slider, so the processing error and positioning error must be strictly controlled during processing. Additionally, it is very important to reasonably arrange the steps in the processing procedure. The microspring was fabricated through a combination of multiple cutting and destress annealing steps. Although the flexural deformation of the microspring was significantly improved, deformation still existed. If the “microspring before the V-shaped groove” processing procedure is followed, the positional accuracy of the V-shaped groove cannot be guaranteed. Therefore, the “V-shaped groove-microspring-frame” procedure was adopted during processing, and the processed sample is shown in Figure 24.

### 5.5. Testing the Improved Setback Arming Device

**Figure 25:**
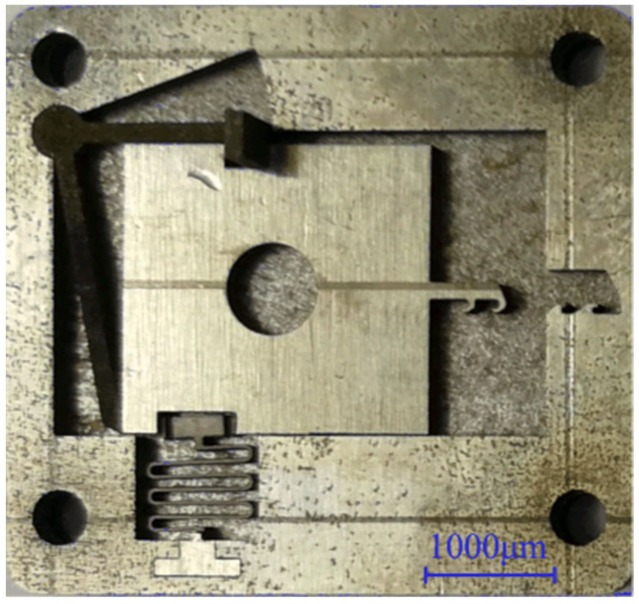
Improved principle prototype.

Figure 25 shows the improved principle prototype. Compared with the original structure, the bottom width of the frame is increased by 1.1 mm. 10 improved principle prototypes are taken as one group, and a total of 100 groups of mechanical impact tests and centrifugal overload tests are carried out under the same test conditions as those in Section 4 of this paper.

#### 5.5.1. Mechanical Impact Test Results of Group 1

**Figure 26:**
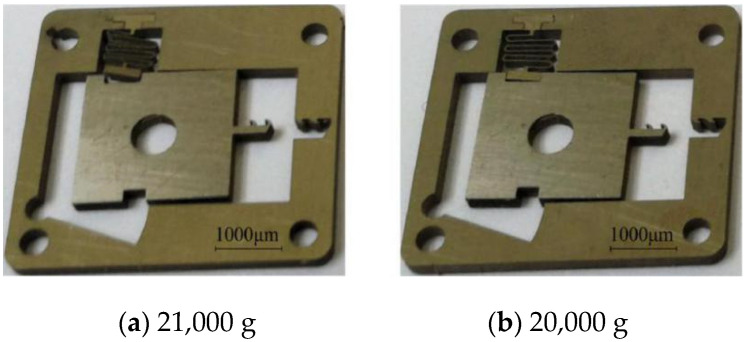
Mechanical impact test results.

The results of the mechanical impact test of group 1 are shown in Table 8. When the peak acceleration decreases from 23,000 g to 22,000 g, the angle of the V-shaped groove at both ends of the setback slider increases after it completely moves away from the arming slider. The setback slider then gets stuck on the arming slider and cannot return to the initial position. When the peak acceleration decreases to 21,000 g, the angle of the V-shaped groove at both ends of the setback slider decreases, and some inclined planes break away from the arming slider and cannot return to the initial position. When the peak acceleration decreases from 20,000 g to 14,000 g, the setback slider remains in the initial position, and the angles of the V-shaped grooves at both ends do not change. Figure 26 includes the scanning electron microscopy (SEM) images of the setback arming device when the peak acceleration is 21,000 g and 20,000 g.

#### 5.5.2. Centrifugal Overload Test Results of Group 1

**Figure 27:**
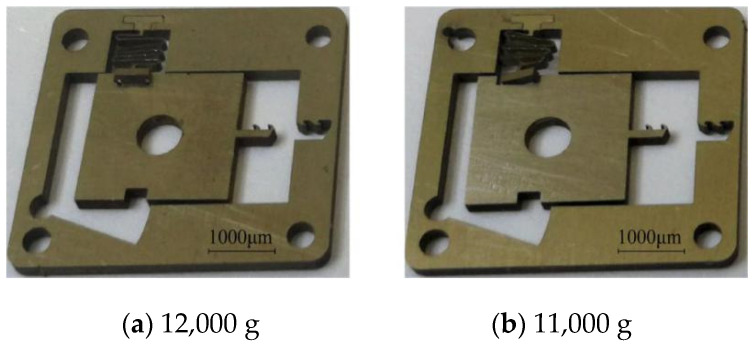
Centrifugal overload test results.

The results of the centrifugal overload test of group 1 are shown in Table 9. When the centrifugal acceleration decreases from 17,000 g to 12,000 g, the setback slider can move to the fully released position. When the centrifugal acceleration decreases to 11,000 g, the angles of the V-shaped grooves at both ends of the setback slider decrease, and some inclined planes break away from the arming slider, but the safety is not completely released. When the centrifugal acceleration decreases from 10,000 g to 8000 g, the setback slider always remains in the initial position and cannot be released. Figure 27 shows the SEM images of the setback arming device when the centrifugal acceleration is 12,000 g and 11,000 g.

#### 5.5.3. Statistics of Test Results

**Figure 28:**
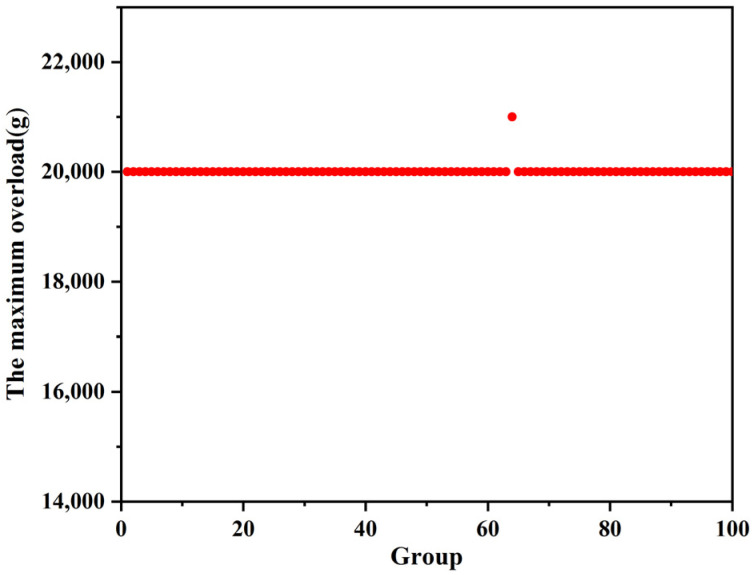
Mechanical impact test results.

**Figure 29:**
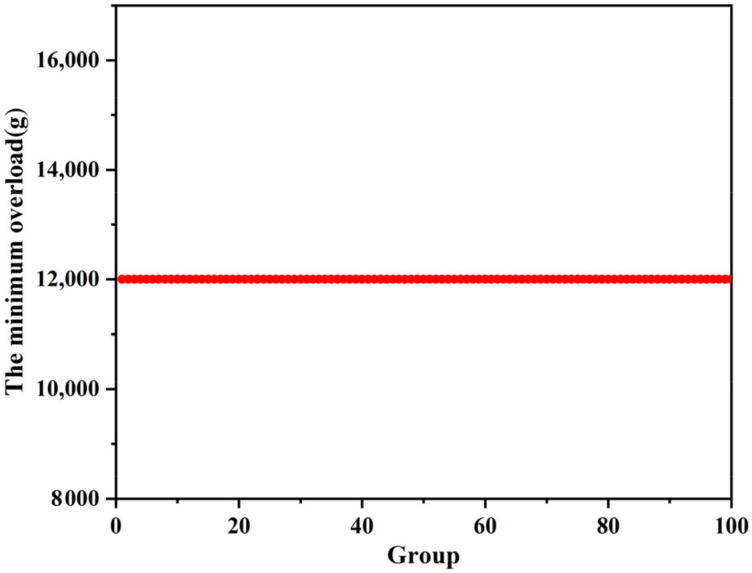
Centrifugal overload test results.

Figure 28 shows the maximum overload that the improved setback arming device can be withstood in the mechanical impact test, and Figure 29 shows the minimum overload that the improved setback arming device for safety release in the centrifugal overload test. Table 10 shows the statistical results. It can be seen from the table that the test results of the two groups fall within the tolerance range, which satisfies the “3σ criterion”. Taking into account the influence of machining errors and test conditions, the statistical results show that the improved setback arming device can guarantee the safety of service processing and the reliability of launch. The maximum overload that can be withstood in service processing is 20,000 g, and the minimum overload for safety release during launch is 12,000 g.

## 6. Conclusions

In this paper, the EDM process is successfully applied to the fabrication of a vertical-frame-based setback arming device, and a mechanical impact test and centrifugal overload test are carried out on the principle prototype. The structure of the principle prototype is then improved according to the problems indicated by the test, and the conclusions are as follows:

1. The setback arming device fabricated by the EDM process can greatly reduce the machining cost while improving the machining precision and machining speed, which can compensate for the defects of the current manufacturing technology. The problems of flexural deformation and secondary deformation in the microspring processing can be effectively solved by designing the auxiliary support beam, using multiple cuts, destress annealing, and optimizing the processing parameters. When the width of the auxiliary support beam is 0.04 mm and the position is set at the center of the microspring end, the influence on the microspring during processing is minimized. At this point, the numbers of gears that are selected for the machining tool are pulse interval SB = 18, peak current IP = 3, flushing pressure LQ = 5, reference voltage VG = 75, electrode wire tension WT = 4, and wire speed WS = 9.
2. The difficult deformation problem can be effectively solved by establishing V-shaped grooves at both ends of the setback slider. The distance between the vertical sidewall of the V-shaped groove and the inclined plane is 0.25 mm, and the distance between the vertical sidewalls is 1.5 mm. The vertical sidewall has a depth of 0.35 mm and a sharp angle of 16°.
3. The setback arming device connected by a clearance fit between the microspring and the frame can not only be used to simplify the fabrication process and minimize testing costs but also guarantees the service processing safety and launch reliability. The maximum overload that can be withstood in service processing is 20,000 g, and the minimum overload for safety release during launch is 12,000 g.

## Figures and Tables

**Table 1:** Process comparison.

Parameter	UV-LIGA Process	EDM Process
Perpendicularity error	≤5%	≤0.3%
Machining time	≤640 min	≤5 min
Machining error	≤10%	≤1%
Machining cost	High	Very low

**Table 2:** Optimized processing parameters.

Parameter	Pulse Interval SB	Peak Current IP	Flushing Pressure LQ	Discharge Energy SA	Reference Voltage VG	Wire Speed WS	Wire Tension WT
Value ^1^	18	3	5	2	75	9	4

^1^ The values in the table are numbers of machine tool gears, not actual values.

**Table 3:** Unilateral discharge gap.

Parameter/μm	*a*_1_	*a*_2_	*a*_3_	*a*_4_	*a*_5_
Value	21	16	12	9	7

**Table 4:** Electrode wire offset.

Parameter/μm	*M*_1_	*M*_2_	*M*_3_	*M*_4_	*M*_5_
Value	121	92	73	62	57

**Table 5:** Main parameters of the processing electrode.

Processing Sequences	Wire Compensation/ μm	Cutting Speed mm/min	Reference Voltage	Peak Current
1	181 ^1^	5	50	9
2	131	4	100	8
3	116	3	80	4

^1^ The values in the table are numbers of machine tool gears, not actual values.

**Table 6:** Forming dimensions of the electrode.

Electrode	Height *h*′/mm	Angle *α*′/(°)	Distance between the Two Electrodes *l*′/mm
Left	0.59	16.1°	1.52
Right	0.61	16.2°	

**Table 7:** Forming dimensions of the V-shaped groove.

V-Shaped Groove	Depth *h*/mm	Angle *α*/(°)	Distance between the Vertical Sidewalls *l*/mm
Left	0.347	16.2°	1.51
Right	0.352	16.4°	

**Table 8:** Mechanical impact test results of group 1.

Sample Number	Peak Acceleration/g	Setback Slider Position
#1	23,000	The angle of the V-shaped groove increases and it gets stuck on the arming slider.
#2	22,000	The angle of the V-shaped groove increases and it gets stuck on the arming slider.
#3	21,000	The angle of the V-shaped groove decreases and some inclined planes break away from the arming slider.
#4	20,000	Initial position
#5	19,000	Initial position
#6	18,000	Initial position
#7	17,000	Initial position
#8	16,000	Initial position
#9	15,000	Initial position
#10	14,000	Initial position

**Table 9:** Centrifugal overload test results of group 1.

Sample Number	Centrifugal Acceleration/g	Setback Slider Position
#1	17,000	The angle of the V-shaped groove increases and it gets stuck on the arming slider.
#2	16,000	The angle of the V-shaped groove increases and it gets stuck on the arming slider.
#3	15,000	The angle of the V-shaped groove increases and it gets stuck on the arming slider.
#4	14,000	The angle of the V-shaped groove increases and it gets stuck on the arming slider.
#5	13,000	The angle of the V-shaped groove increases and it gets stuck on the arming slider.
#6	12,000	The angle of the V-shaped groove increases and it gets stuck on the arming slider.
#7	11,000	The angle of the V-shaped groove decreases and some inclined planes break away from the arming slider.
#8	10,000	Initial position
#9	9000	Initial position
#10	8000	Initial position

**Table 10:** Statistical results.

Group	Average Value/g	Standard Deviation/g	Minimum Value/g	Maximum Value/g	Tolerance Range/g
Mechanical impact test	20,010	99.50	20,000	21,000	19,711.5~20,308.5
Centrifugal overload test	12,000	0	12,000	12,000	12,000

## References

[1] Li M., Hu T. (2021). Research Status and Development Trend of MEMS S&A Devices: A review. Def. Technol..

[2] Fan L., Last H., Wood R., Dudley B., Malek C., Ling Z. (1998). SLIGA Based Underwater Weapon Safety and Arming System. Microsyst. Technol..

[3] Zhang H., Wang Y., Lou W., Liu F., Wang F. (2014). Reliability Factors Analysis of MEMS Safety and Arming System. Key Eng. Mater..

[4] Wang F., Lou W., Fu Y., Wang Y. Parametric Research of MEMS Safety and Arming System. Proceedings of the 8th Annual IEEE International Conference on Nano/Micro Engineered and Molecular Systems.

[5] Nie W., Xi Z., Xue W., Zhou Z. (2013). Study on Inertial Response Performance of A Micro Electrical Switch for Fuze. Def. Technol..

[6] Swati R., Suryaprakash, Duttagupta S., Mutthurajan H. MEMS Based Spin Sensor Displacement Analysis in Nanometer Precision Using COMSOL for S&A Devices. Proceedings of the 2016 3rd International Conference on Devices, Circuits and Systems (ICDCS).

[7] Michael D., Peter S., Rajesh S. Packaging of a MEMS Based Safety and Arming Device. Proceedings of the ITHERM 2000, the Seventh Intersociety Conference on Thermal and Thermomechanical Phenomena in Electronic Systems (Cat. No.00CH37069).

[8] Wang F., Lou W., Xiong Y., Liu F., Wang D., Jin X., Zhang M., Xu C. The Parametric Analysis of the Centrifugal Insurance Mechanism in MEMS Safety and Arming Device. Proceedings of the 10th IEEE International Conference on Nano/Micro Engineered and Molecular Systems.

[9] Robinson H. (2012). Ultra-Minature Electro-Mechancal Safety and Arming Device. U.S. Patent.

[10] Rawata L., Kadama S., Yadava K., Bhandarib R., Kumarc V. (2015). MEMS Based Safe Arm and Fire Device (SAM). Int. J. Innov. Emerg. Res. Eng..

[11] Pezous H., Rossi C., Sanchez M., Mathieu F., Dollat X., Charlot S., Salvagnac L., Conédéra V. (2010). Integration of a MEMS based safe arm and fire device. Sens. Actutors A Phys..

[12] Pezous H., Rossi C., Sanchez M., Mathieu F., Dollat X., Charlot S., Conedera V. (2010). Fabrication, assembly and tests of a MEMS-based safe, arm and fire device. J. Phys. Chem. Solids.

[13] Zhang Y., Lou W., Wang D., Liao M. (2019). Design and reliability analysis of multi-scale security system for Microminismart ammunition. Microsyst. Technol..

[14] Xie R., Ren X., Liu L., Xue Y., Fu D., Zhang R. (2014). Research on Design and Firing Performance of Si-based Detonator. Def. Technol..

[15] Pennarun P., Rossi C., Estève D., Bourrier D. (2006). Design, fabrication and characterization of a MEMS safe pyrotechnical igniter integrating arming, disarming and sterilization functions. J. Micromech. Microeng..

[16] Seok J., Jeong J., Eom J., Lee S., Lee C., Ryu S., Oh J. (2017). Ball Driven Type MEMS SAD for Artillery Fuse. J. Micromech. Microeng..

[17] Jeong J., Eom J., Lee S., Lim D., Jang Y., Seo K., Choi S., Lee C., Oh J. (2018). Miniature Mechanical Safety and Arming Device With Runaway Escapement Arming Delay Mechanism for Artillery Fuze. Sens. Actutors A Phys..

[18] Sun D., Tang Y., Wang J., Wang X. (2020). Design of an H-shaped Linear Piezoelectric Motor for Safety and Arming Device. Sens. Actutors A Phys..

[19] Wang K., Hu T., Zhao Y. (2021). A MEMS Inertial Driven Runaway Escapement Mechanism in Fuze. J. Detect. Control.

[20] Li X., Zhao Y., Hu T., Xu W., Zhao Y., Bai Y., Ren W. (2015). Design of a Large Displacement Thermal Actuator with a Cascaded V-Beam Amplification for MEMS Safety and Arming Devices. Microsyst. Technol..

[21] Koehler (2006). Microelectromechanical Safing and Airming Apparatus. U.S. Patent.

[22] Li W. (2018). Research on Key Technologies of MEMS Safety System. Master’s Thesis.

[23] Tong X., Nie W., Xi Z. (2019). The Design of the Setback Arming Mechhanism for MEMS Security System. Mach. Electron..

[24] Du L., Jia S., Nie W., Wang Q. (2011). Fabrication of Fuze Micro-electro-mechanical System Safety Device. Chin. J. Mech. Eng..

[25] Du L., Wang A., Zhao M., Song M. (2014). The Fabrication of Trans-scale Micro-fuze Safety Device. Key Eng. Mater..

[26] Sanchez J., Plaza S., Lopez De Lacalle L., Lamikiz A. (2006). Computer Simulation of Wire-EDM Taper-cutting. Int. J. Comput. Integr. Manuf..

[27] Sanchez J., Lopez De Lacalle L., Lamikiz A. (2004). A Computer-aided System for the Optimization of the Accuracy of the Wire Electro-discharge Machining Process. Int. J. Comput. Integr. Manuf..

[28] Abdallah R., Soo S., Hood R. (2021). The influence of cut direction and process parameters in wire electrical discharge machining of carbon fibre-reinforced plastic composites. Int. J. Adv. Manuf. Technol..

[29] Schaller T., Bohn L., Mayer J., Schubert K. (1999). Microstructure Grooves with a Width of Less Than 50 mm Cut with Ground Hard Metal Micro End Mills. Precis. Eng..

[30] Fonda P., Katahira K., Kobayashi Y., Yamazaki K. (2012). WEDM Condition Parameter Optimization for PCD Microtool Geometry Fabrication Process and Quality Improvement. Int. J. Adv. Manuf. Technol..

[31] Li Y. (2019). Experimental Research on Machining Micro Serpentine Spring by EDM. Master’s Thesis.

[32] Fan D., Xu Y., Tong X. (2011). Heat Treatment Engineer’s Manual.

[33] Rajarshi M., Shankar C., Suman S. (2012). Selection of Wire Electrical Discharge Machining Process Parameters Using Non-traditional Optimization Algorithms. Appl. Soft Comput..

[34] Gill R., Kumar K., Batra U. (2021). Surface Characteristics and Corrosion Behavior of Wire Electrical Discharge Machining Processed Mg-4Zn Alloy. J. Mater. Eng. Perform..

